# A pilot study of patient satisfaction with a self-completed tablet-based digital questionnaire for collecting the patient’s medical history in an emergency department

**DOI:** 10.1186/s12913-021-06748-y

**Published:** 2021-07-30

**Authors:** Leander Melms, Juergen R. Schaefer, Andreas Jerrentrup, Tobias Mueller

**Affiliations:** 1 Center for undiagnosed and rare diseases, University Hospital Gießen and Marburg, 35033 Marburg, Germany; 2 Institute of Artificial Intelligence, Philipps-University Marburg, 35033 Marburg, Germany; 3 Emergency Department, University Hospital Gießen and Marburg, 35033 Marburg, Germany

**Keywords:** Self-administered questionnaires, Tablet computers, Patient-reported outcome, Anamnesis, Survey, Tablet, Tablet computers, Self-assessment, Patient-conducted computer interview, Computerized questionnaires; self-anamnesis

## Abstract

**Background:**

The increasing popularity and availability of tablet computers raises questions regarding clinical scenarios. This pilot study examined the patient’s satisfaction when using a tablet-based digital questionnaire as a tool for obtaining medical history in an emergency department and to what extent gender, age, technical competence and mother tongue influence the user satisfaction. Patients were asked to complete three consecutive questionnaires: The first questionnaire collected basic epidemiological data to measure past digital usage behaviour, the second questionnaire collected the patient’s medical history, and the third questionnaire assessed the overall perceived user satisfaction when using the tablet-based survey application for medical anamnesis.

**Results:**

Of 111 consenting patients, 86 completed all three questionnaires. In summary, the user evaluation was positive with 97.7% (*n* = 84) of the patients stating that they had no major difficulties using the digital questionnaire. Only 8.1% (*n* = 7) of patients reported a preference to fill out a paper-and-pen version on the next visit instead, while 98.8% (*n* = 85) stated that they would feel confident filling out a digital questionnaire on the next visit. The variables gender, age, mother tongue and/or technical competence did not exert a statistically significant influence towards the defined scales usability, content and overall impression.

**Conclusion:**

In conclusion, self-administered tablet-based questionnaires are widely accepted tools for collecting medical information in the emergency room across all ages and genders, regardless of technical competence.

**Supplementary Information:**

The online version contains supplementary material available at 10.1186/s12913-021-06748-y.

## Introduction

The assessment of a comprehensive, problem-based medical history is decisive for further goal-oriented diagnostics and has a major influence on the therapy recommendation. Prior studies have identified four main problem areas as critical sources of error in the medical anamnesis [1]: (i) A coherent and concise presentation of the medical history in chronologically correct order is strongly related to the understanding of the patient’s clinical complaints; (ii) face-to-face fear or embarrassment during the patient interview can result in a misleading clinical assessment; (iii) medical terminology and interruptions on the part of the clinician can – due to intimidation or confusion – cause an incomplete description of the problem; (iv) there may be a practitioner bias based on gender, race and/or culture that may result in inappropriate variations in questions and answers and may be an obstacle to obtaining a detailed medical history. These factors are of even greater importance in the emergency department, since there is usually no previously created doctor-patient relationship in this environment, and decisions about medical interventions must be made under great time pressure [1].

An innovative approach to self-anamnesis is the use of tablet-based questionnaires for non-urgent patients in the emergency department. In the e-health age, we add to the body of evidence concerning using a tablet-based questionnaire in the emergency department in routine operations of an university clinic by focusing on the influence of age, gender and/or technical competence towards the overall satisfaction, usability and understanding of the content of the questionnaire. As far as we know, previous studies have directly measured the overall satisfaction, usability and comprehension of the content of the digital questionnaire with corresponding items. However, we conceived the variables to be measured as constructs or latent variables and have tried to measure them in as many dimensions as possible. For this purpose, the three previously “formed” scales “usability”, “overall satisfaction” and “understanding of content” were established. To the best of our knowledge, the applied questionnaire exceeds previous evaluations of user satisfaction not only quantitatively but also qualitatively. We were able to demonstrate that the previously positive results are also reflected in a comprehensive evaluation questionnaire.

## Literature review

### Benefits

The main benefits of digital questionnaires include the improved data quality, the data quantity [2–4] and the facilitation of direct computerized processing without media breaks. Using standardized questionnaires, more details regarding the patient’s medical history can be collected than through doctor’s consultation alone [2–4]. The quantity of information collected using a digital medical history device corresponds – or in some cases partially exceeds – the amount of information that can be derived from a personal conversation [5, 6]. In addition, several studies have indicated that sensitive or even “delicate” personal information can be better collected using (computer-based) questionnaires: Patients were more sincere when using computer-assisted questionnaires than in doctor-patient interviews [7], for instance in the case of collecting HIV risk factors [4, 8, 9]. In a study conducted by Arora et al. over 83% of patients stated that they preferred to answer sensitive questions using a digital questionnaire rather than in person [1].

From a technical perspective previous work has demonstrated that around 85–93% of adult study participants experience no technical problems when using a digital questionnaire [10, 11]. The evaluation of the user friendliness of this format showed that around 97% of the patients would prefer to fill out a digital questionnaire in the future. About 94% of patients felt that the digital questionnaire would help structure their thoughts and would ultimately have a positive effect on treatment [1]. Previous research has also found that patients, particularly those under the age of 50, favour digital questionnaires over paper-and-pen versions [12, 13]. In a review by Dale et al. the authors were able to demonstrate that digital questionnaires outperform conventional paper questionnaires in the categories of feasibility, user satisfaction and data accuracy [12]. This finding was further confirmed in a study by Hauber et al.: Patients of a pulmonary outpatient clinic were given a dynamic multilingual questionnaire and over 80% of the participants found the tool to be more convenient compared to paper-and-pen. In addition, 93% found the tool to be “helpful” or “rather helpful” and 89% saw an advantage in completing the questionnaire before talking to the physician [14]. The overall highly positive impression of digital questionnaires has been demonstrated in numerous studies [14–21]. Herrick et al. were able to show that less than 1% of patients would describe the digital questionnaire as difficult to complete. Most patients (86%) did not require support, and those patients requesting help were older on average (54 ± 19 vs. 40 ± 15 years) [22]. In the Benaroia study, 85% of respondents said they found the digital medical history to be easy to use, and over 92% reported a willingness to fill out a digital questionnaire again [23]. The level of satisfaction has been even exceeded in the study by Arora et al., were over 97% of patients stated that they would like to fill out a digital questionnaire again in the future [1]. The advantage of saving time is often cited in the literature (e.g. in the review by Bachman et al.). This benefit would result from the fact that the documentation effort could be reduced, and accordingly more time could be scheduled for the patient. However, evidence supporting this outcome is scarce [12]. To the best of our knowledge, only one study has measured an actual time saving of 15 min per patient [24].

### Risks

The risks of implementing a (digital) questionnaire concern the amount of information and the accuracy of the data collected. Although the information is described by the treating physicians as comprehensive, it is too detailed for simpler medical issues [6]. Moreover, the accuracy of the medical history data has been described as problematic in some cases [5]. In the study by Zakim et al., serious false-negative answers were found. For example, long-term diabetics stated that they did not suffer from diabetes mellitus, while patients with confirmed coronary heart disease stated that they did not suffer from any kind of stress-related angina pectoris. The authors attributed this discrepancy to inadequately formulated questions and a lack of medical understanding on the part of the patients [5]. Technical problems in routine use should also not be neglected. For instance, in the review by Dale et al., a data loss due to software and hardware errors was described [12]. Furthermore, any additional costs must be calculated. Dale et al. pointed out, in addition to the system itself, technical personnel may also have to be hired [12]. Moreover, besides the technical implementation, strategies must be developed to prevent theft or damage to the equipment.

## Methods

### Place of study and recruitment

The pilot study was conducted in the waiting area of the central interdisciplinary emergency department of the Marburg University Hospital. Study participants were recruited randomly by personal contact in the waiting area after the initial emergency triage and before first contact with the physician (convenience sampling). Recruitment was performed by a single researcher (LM) and took place from July 2017 to July 2018. No expense allowance was handed out, and participation in the study was voluntary. Patients with the following characteristics were excluded: aged under 18 years; psychological restrictions that precluded an understanding of the questionnaire or the study information; physical limitations that did not allow for the use of the digital questionnaire (e.g. blindness, missing/limited finger motor skills); medically urgent treatment as indicated by triage higher than green according to the Manchester triage system (MTS). Prior to enrolment, the admission protocol and the information obtained by the nursing staff were used to ensure that full legal capacity existed and that no medical assistance service was arranged. If during the process of administrative admission, triage, informed consent and completion of the tablet-based questionnaire, the patient indicated or gave the impression that they were experiencing difficulties in participation (e.g. dementia, acute alcoholization), the patient was not included in the pilot study.

### Technology and data collection procedure

**Fig. 1:**
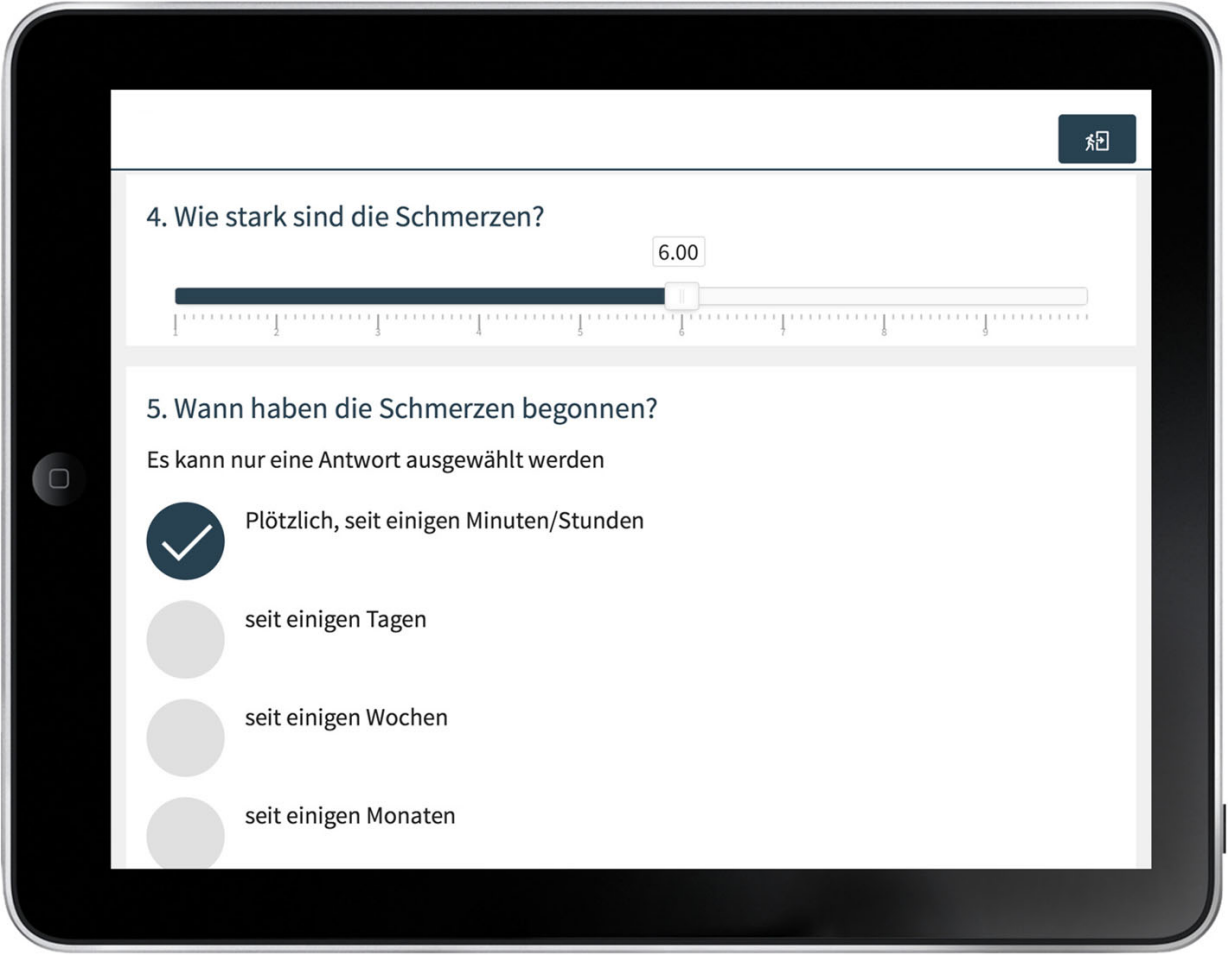
Screenshot, illustrating the display of the questionnaire application used

The software utilized for displaying and collecting the patient questionnaires was MyMedax provided by Suxedo, Saarbrücken, Germany. The software was completely hosted on premise, on a dedicated server (Ubuntu 14.04 with MySQL 5.7 database) within the network of the Marburg University Hospital. In terms of data protection, a consecutive number was used for identification and no data were cached on the device. Only application-internal data (information on connectivity, etc.) were stored on the iPad tablets (Apple Inc., Cupertino). The graphical user interface to answer the questions contained free-text input fields, single-choice and multiple-choice questions, date fields and slider types. The free-text input fields could be configured so that only a certain (text) input was possible. For instance, in the case of the item that collected the age of the respondents, only numeric integers were accepted. Conversely, the item that recorded the mother tongue data accepted any input information. A sample Screenshot of the user interface can be seen in Fig. 1.

### Patient questionnaires

Included and consenting patients were briefly instructed in the use of the tablet/software and were asked to answer three distinctive questionnaires in a fixed order: The first questionnaire collected basic epidemiological data; the second recorded the patient’s medical history and presenting complaints; the third and final questionnaire assessed the user experience (see supporting material for details). Patients were asked to report as soon as they had finished the task, whereupon they returned the tablet back to the researcher.

### First questionnaire – basic epidemiological data

This questionnaire was used to record epidemiological parameters (age, gender, etc.) and data about each patient’s private use of digital devices. Information regarding visual impairments and motor impairments which could make it difficult to fill in the questionnaire on the tablet was also collected (see Table 1).

**Table 1:** Items of the basic epidemiological questionnaire I

Variable	No.	Question	Response options
Age	1	How old are you?	Natural numbers without zero [Numerical input]
Gender	2	Gender?	male, female, not specified [Single Choice]
Mother tongue	3	Mother tongue?	Free text input field
Digital device usage	4	Which devices do you regularly use privately?	Mobile phone, smartphone, laptop, stationary computer, none of these [Multiple Choice]
Computer skills	5	I consider my computer skills as very good	4-point Likert (Strongly agree – strongly disagree) [Single Choice]
Visual impairment	6	Do you need a visual aid (e.g. glasses, contact lenses)?	Yes/No [Single Choice]
	7	Are you suffering from any of the listed eye diseases?	Selection of vision-limiting eye diseases (Colour blindness, Macular degeneration, Glaucoma, Cataract, Diabetic retinopathy, None) [Multiple Choice]
Motor limitations	8	Do you have any of the listed motor limitations?	Selection of fine motor skills impairing diseases (o Parkinson syndrome, Disease of the rheumatic form, Amputations of fingers, Numbness of the fingers, Other, None) [Multiple Choice]

### Second questionnaire – medical history

The second questionnaire consisted of a basic anamnesis, including questions to collect information on presenting complaints, risk factors, allergies, previous illnesses and medication. Only the German language was used. The wording of the questions was taken from the literature, from existing anamnesis forms and from medical consent forms to facilitate simple language and a high level of comprehensibility. The questionnaire was coordinated with the senior physicians of the departments involved. The questionnaire for the internal medicine department contained 137 individual questions; the questionnaire for the surgical and trauma department contained 47 individual questions (the surveys are provided in the supporting material). The user satisfaction was not compared based on the type of the questionnaire filled.

### Third questionnaire – user experience

After their medical history was obtained, the study participants were asked to briefly evaluate the user experience. This questionnaire was utilized to record the essential characteristics of technical competence / overall impression and the understanding of the content, data protection and overall impression. All items, except item 27, were recorded on a 4-point Likert scale (“strongly agree”, “agree”, “rather disagree”, “strongly disagree”). Item 27, “How satisfied were you with your answers to the digital questionnaire?”, collected a point value between 0 and 10 for an initial assessment of the global user experience using a ratio scale (implemented with a range slider control). This question was not included in the construct “usability”.

The items of the scale “usability” are mainly taken from the literature and from the sufficiently validated System Usability Scale (SUS) [25]. The SUS questionnaire served as content orientation for the formulation of items here, and the translation was not strict and was not validated. The items for the scale “understanding of content” were newly formulated by the working group in consensus. According to Fisseni [26], the following principles were taken into account in the construction of the questionnaire and formulation of the items:

- The formulation of the item should be oriented “...to the everyday (colloquial) language of the average member of the target population.”
- The sentences “...should be short and rarely exceed 20 words.”
- The items should be balanced, that is, one part of the answers should be positive, the other part negative in the key direction.

The survey items were not grouped by the defined scales but instead displayed in a fixed order. The assignment of the items to the scales is shown in Table 2.

**Table 2:** Questions of the third survey

Scale	No	Question	Score
Usability 15 Questions	27	How satisfied were you with the usability of the digital questionnaire?	8.79 ± 1.34
	19	I was able to complete the questionnaire in peace.	2.95 ± 0.21
	13	I felt confident to be able filling out the digital questionnaire.	2.86 ± 0.35
	06	The answers to the questions worked perfectly from a technical point of view.	2.88 ± 0.39
	07	The time to complete the questionnaire was just right.	2.81 ± 0.42
	25	I was completely satisfied with the colour representation of the digital questionnaire.	2.79 ± 0.63
	20	I always knew exactly where to click to answer the questions.	2.65 ± 0.73
	16	* I was afraid to damage / drop the device.	2.62 ± 0.86
	11	The font was legible.	2.99 ± 0.11
	12	* The font size was too small.	2.83 ± 0.60
	01	* The tablet was difficult for me to use.	2.83 ± 0.58
	04	* The questionnaire sometimes reacted slowly.	2.84 ± 0.57
	10	The handling of the device was easy for me.	2.88 ± 0.32
	14	I feel confident to be able filling out another digital questionnaire in the same format.	2.87 ± 0.37
	18	* I would rather have filled out a paper and pen version of the questionnaire.	2.59 ± 0.80
Content 8 questions	05	The questionnaire was clearly structured.	2.79 ± 0.51
	17	I had no problems understanding the questions, even if there were medical terms.	2.38 ± 0.94
	08	* I had difficulty in understanding the questions.	2.40 ± 0.97
	21	I always knew what the questions were about.	2.62 ± 0.62
	26	Unknown terms, if any, were explained.	2.62 ± 0.74
	09	The questions seemed appropriate and conclusive to me.	2.83 ± 0.51
	03	The questionnaire helped to present my medical concerns.	2.44 ± 0.78
	15	I had the impression that the questionnaire helped to sort my thoughts.	1.63 ± 1.12
Privacy 1 question	02	I rate the data protection and data security of the questionnaire as high.	2.40 ± 0.71
Overall impression 3 questions	24	My overall impression is quite positive.	2.68 ± 0.63
	23	* I would not recommend the digital questionnaire.	2.56 ± 0.86
	22	I would like to have a digital questionnaire again in the future.	2.48 ± 0.84

Negatively formulated items were (01, 04, 08, 12, 16, 18, 23) transformed and marked in the data record with an asterisk (*) and the points were assigned in reverse order (x - 3), so that a high expression of the items also indicates a higher level of agreement. Questions are ordered by appearance. Scores are reported as average ± standard deviation

### Data analysis

#### Data preparation and data management

All data were obtained from a database query (SQL) in comma-separated values format from the questionnaire server. The program libraries pandas [27], SciPy [28], Matplotlib [29], seaborn [30] and Jupyter [31] were utilized for exploratory data analysis, data post-processing and plotting.

#### Statistical evaluation

Basic data were analysed using descriptive statistics. Time to complete was measured using the technical logfiles as the difference between the start and end of the questionnaire. For analysis, the Likert questionnaire items were assigned a corresponding point value between 0 and 3 (0 = strongly disagree, 1 = rather disagree, 2 = rather agree, 3 = strongly agree) and the centre of the scale is 1.5. Negatively formulated items were transformed, and the points were assigned accordingly (*x* - 3), so that a high level of the item point values also indicated a higher level of approval. This transformation concerned the items 01, 04, 08, 12, 16, 18 and 23; a marking (*) was subsequently added to the data record. An example of the item transformation can be illustrated by item 23: “I would not recommend the digital questionnaire” becomes “I would recommend the digital questionnaire”.

Missing data entries were replaced by the mean value of the characteristic. The average score of each scale (usability, content, overall impression) was calculated by adding up the Likert values of the individual items and dividing by the number of items in the category. Groups were formed based on the independent variables age, gender, mother tongue and technical competence. Prior to the hypothesis test, the epidemiological basic parameters gender and age of the study population were checked for normal distribution using previously obtained triage data from 2016 with the Shapiro-Wilk test. We examined the dependent variable age for normal distribution. The variable gender is a factor here, since we have collected data on age for both male and female subjects and wanted to analyse them separately. The primary targets were then evaluated using non-parametric tests: the Mann-Whitney U test for two groups and the Kruskal-Wallis test for more than two groups. All data are presented as average ± standard deviation, unless otherwise stated. All statistical tests were performed with a significance level of α = 5%.

#### Ethics approval

Ethics approval for the study was obtained from the Philipps-University Medical Ethics Committee (No 66/17). The present study was conducted in accordance with the Declaration of Helsinki and all research was performed in compliance with relevant guidelines and regulations. Written informed consent was obtained from all subjects before their inclusion in the study.

## Results

### Description of the characteristics of the patient collective

A total of 111 patients were asked to participate in the study, two of whom (1.8%) refused. Moreover, 23 (20.7%) patients were unable to complete the medical history form due to interruptions in patient care. These data sets were not considered in the evaluation. Altogether, 86 (77.5%) patients fully completed the questionnaire.

The age of the study group ranged from 19 to 84 years with a median of 41.8 ± 16.0 years. Three age groups were formed: under 30 years (37.2%), between 30 and 60 years (50%), and over 60 years (12.8%). The age distribution of the study collective is not normally distributed (mean 41.59 ± 16.3; Shapiro-Wilk test, *p* = 0.0006) and it is significantly different from the known normally distributed collective from the total number of visits recorded in 2016 (mean 53.4 ± 22.3 years; Shapiro-Wilk test, *p* = 0.95). The subjects were 51.2% male and 48.8% female, with average ages of 38.4 ± 14.1 years and 44.9 ± 17.9 years, respectively.

Of the study participants, 91.9% had previous knowledge concerning the use of digital devices (mobile phone, smartphone, tablet computer, laptop, stationary computer). The study participants were divided on the basis of the item “I rate my computer skills as very good.” into four groups with regard to everyday computer skills: 54.7% of the patients rated their computer skills as very good (strongly agree), 24.4% as good (agree), 11.6% as moderate (rather disagree) and 9.3% as bad (strongly disagree). The mean was 2.3 ± 1.0 points and is therefore between “very good” and “good”. Table 3 provides a summarizes the patients’ collective characteristics.

**Table 3:** Summary of the patient collective characteristics

Characteristic	n	%
Gender		
- male	44	51.2
- female	42	48.8
Age		
- < 30	32	37.2
- 30–60	43	50.0
- > 60	11	12.8
Computer skills		
- Very Good	47	54.7
- Good	21	24.4
- Poor	10	11.6
- Bad	8	9.3
Mother tongue		
- German	74	86.0
- other	12	14.0
Priv. use of digital devices		
- Mobile phone	52	60.0
- Smartphone	19	22.1
- Stationary/fixed computer	5	5.8
- Laptop / Notebook	3	3.5
- None of these	7	8.1
Physical impairments		
visual impairment *		
- None	80	93.0
- Colour blindness	2	2.3
- Diabetic retinopathy	1	1.2
- Macular Degeneration	1	1.2
- Cataract	1	1.2
- No response	1	1.2
motor impairments		
- None	83	96.5
- Numbness of the fingers	1	1.2
- Other	1	1.2
- No response	1	1.2
Incomplete data sets	25	22.5

(*) multiple answers were possible

### Results of the hypothesis tests

The age proved to be significantly different within the groups formed based in respective computer skills (*p* = 0.016, Kruskal-Wallis test). The mean age of the study participants with poor computer skills was 66.50 ± 13.36 years, 50.60 ± 15.01 years for those with moderate computer skills, 43.29 ± 12.19 years for those with good computer skills, and 34.68 ± 13.43 years for those with very good computer skills. Table 4 presents a summary of the relationship between age and computer skills. Gender also had a significant impact on the self-assessment of computer skills (*p* = 0.007, Mann-Whitney *U* test). The average computer knowledge of the female study participants was 2.0 ± 1.1, which corresponds to the group “good”. The mean of the male participants is slightly higher at 2.5 ± 0.9 and corresponds to an assessment between “good” and “very good”. The patients needed an average of 5.7 ± 2.2 min (43 items) to complete the questionnaire from the Clinic for Trauma Surgery (*n* = 62) and 7.9 ± 3.0 s per question. For the emergency questionnaire from the Clinic for Internal Medicine (*n* = 20), the participants required 16.4 ± 11.5 min (137 items), with a corresponding average of 7.2 ± 5.0 s per question. The level of computer skills had a significant impact on the questionnaire completion time (*p* = 0.029, Kruskal-Wallis test). The mean of the time to complete both questionnaires was 7.8 ± 3.6 s (*n* = 86). The basic epidemiological parameters age, gender, mother tongue and physical impairments had no significant influence on the completion time.

**Table 4:** Mean Age and its distribution by computer skills

	n	Age	Min	25%	50%	75%	Max
Very good	47	34.7 ± 13.4	19.0	23.5	29.0	44.5	66.0
Good	21	43.3 ± 12.2	19.0	41.0	46.0	51.0	60.0
Poor	10	50.6 ± 15.0	25.0	42.3	52.5	62.3	67.0
Bad	8	66.5 ± 13.4	39.0	65.3	67.0	75.0	84.0

The research hypothesis that patient groups, categorized according to epidemiological factors, physical restrictions and surrogate parameters of digital usage behaviour for technology differed in terms of the constructs defined usability, content and overall satisfaction could not be confirmed. The mean values of the three scales defined were generally high, as measured on a point scale between 0 and 3, with values between 2.4 and 2.8. Table 5 summarizes the results of the statistical significance tests conducted.

**Table 5:** Statistic of the characteristics of the participants in relation to the defined scales

Characteristic	Usability		Content		Overall Impression	*p*-value
Overall	2.84 ± 0.21		2.44 ± 0.43		2.58 ± 0.61	
Gender						
- male	2.84 ± 0.21	p 0.32	2.37 ± 0.46	p 0.51	2.62 ± 0.56	p 0.28
- female	2.83 ± 0.20		2.51 ± 0.41		2.52 ± 0.67	
Age						
- < 30	2.84 ± 0.22	p 0.43	2.42 ± 0.41	p 0.98	2.54 ± 0.70	p 0.08
- 30–60	2.84 ± 0.20		2.42 ± 0.46		2.57 ± 0.59	
- > 60	2.84 ± 0.19		2.58 ± 0.44		2.67 ± 0.46	
Mother tongue						
- German	2.85 ± 0.19	p 0.72	2.45 ± 0.45	p 0.96	2.61 ± 0.45	p 0.27
- other	2.83 ± 0.16		2.38 ± 0.37		2.57 ± 0.64	
Computer Skills						
- very good	2.84 ± 0.21	p 0.12	2.43 ± 0.41	p 0.072	2.63 ± 0.61	p 0.98
- good	2.84 ± 0.22		2.52 ± 0.27		2.60 ± 0.51	
- moderate	2.85 ± 0.21		2.34 ± 0.69		2.50 ± 0.85	
- bad	2.81 ± 0.21		2.36 ± 0.59		2.38 ± 0.63	

**Fig. 2:**
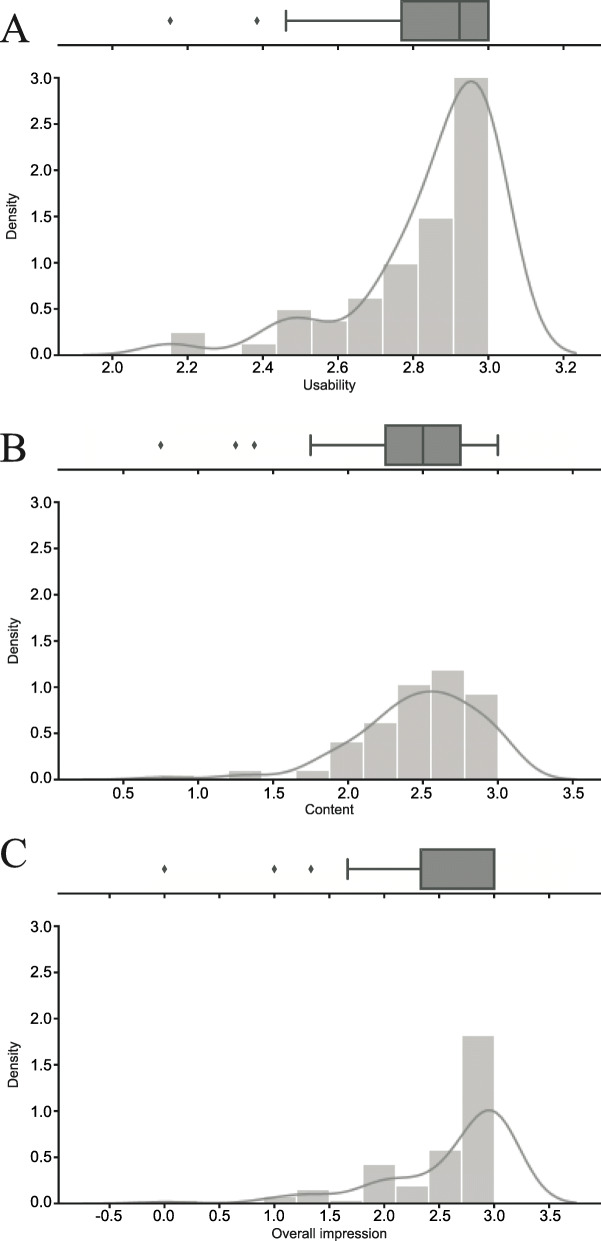
Distribution of the scores of the defined scales. Legend: y-axis: Probability density function for the (Gaussian) kernel density estimation (KDE); x-axis: total score in points (0 = disagree, 1 = rather disagree, 2 = rather agree, 3 = agree). The KDE is a non-parametric technique to estimate the distribution of a variable. A density estimator is an algorithm which seeks to model the probability distribution of the data. The grey line represents a smoothed, continuous estimation of the distribution of the data. In this method, a continuous curve is drawn at every individual data point and all of these curves are then added together to generate a single smooth density estimation. The kernel used is a Gaussian (which produces a Gaussian bell curve at each data point). Figure 2 was generated with the Python 3 library seaborn [30]

Figure 2 shows the graphical representation of the sum scores for each of the defined scales (“usability”, “content”, “overall impression”). The response distribution of all items was strongly left skewed. No mean value was in the negative range (< 1.5 points). In most cases, the complete response range of the 4-point Likert scale was not used. The standard deviations turned out to be small overall, with the ability to differentiate therefore being low overall. A left-skewed distribution can be seen for all 3 scales, corresponding to the mean values.

Overall, 97.7% of the patients stated that they had no difficulties using the digital questionnaire. After answering the questions, 98.8% stated that they would feel confident filling out another digital questionnaire in the same format on the next visit. Only a total value of 8.1% of patients said that they would rather have filled out a paper sheet. No statistically significant gender-, mother tongue- or age-specific influence could be determined (all *p* > 0.05). However, compared to younger patients, patients over the age of 60, with 21%, were more likely not to always know precisely where to click to answer the questions compared to younger patients. Only 9% of patients under the age of 30 had difficulties with task.

In sum 84.9% of the patients agreed that they understood the content of the medical questions, with 59.3% stating that they fully agreed and 25.6% stated that they rather agreed. In terms of serious medical consequences due to inaccurate information, this result should be assessed as questionable. Again, an age-, mother tongue- or gender-specific difference regarding the understanding of the medical content was not observed. Moreover, 87.2% of patients stated that the questionnaire captured their medical concerns fully. Among all patients, 93.0% of the patients had an overall positive impression after completing the digital questionnaire, and 87.2% reported they would prefer to use a digital questionnaire again.

### Privacy

Regarding data protection, 48.8% of the patients fully agreed with the item “I rate the data protection and data security of the questionnaire as high”, 42.9% partially agreed, and only 8.4% said they were concerned.

## Discussion

The study assessed whether a digital questionnaire is a suitable instrument for patient self-anamnesis in the waiting area of an emergency department. In particular, the focus was on the influence of variables such as gender, age and technical competence on the user satisfaction of the digital questionnaire. Previous work in the field of digital anamnesis has reached to a positive conclusion regarding overall impression, usability and satisfaction. This outlook corresponds with the results of this work. Regarding the assessment of user-friendliness, Koch et al. have shown that 80.25% of the participants in their study rated the usability of the applied digital questionnaire as “very good”, 19.75% as “satisfactory” and 1.25% as “unsatisfactory” [17]. In our study, 91.9% of the patients stated that they fully agreed and 5.8% that they rather agreed that the tablet was easy to use. Only 2.3% of patients expressed concerns in this regard. In line with this result, further studies found that 85–93% of the patients experienced no technical issues while handling the digital questionnaire [10, 11].

Regarding the assessment of the overall impression, Arora et al. found that over 97% of patients reported that they would like to fill out a digital questionnaire again in the future. We have been able to confirm this preference in our study: 62.8% of participants fully agreed and 24.4% rather agreed with the statement that they would like to fill out a digital questionnaire again in the future. Only 13.1% of the patients said that they would rather not to do so. The positive overall impression is also expressed in the patients’ preference for choosing a digital questionnaire in contrast to a paper-and-pen version when asked. Hauber et al. demonstrated that over 80% of the participants found the digital questionnaire to be more convenient than paper and pen [14]. In our study, only 3% of the under-30s, 10% of the 30–60s and only 14% of the over-60s stated they would rather have completed the anamnesis using a sheet of paper instead. Furthermore, 91% of those under 30, 95% of those between 30 and 60 and 93% of those over 60 would recommend the digital questionnaire to others.

Satisfaction and confidence in data protection were also measured by Koch et al.: 71.25% rated data protection as high, 21.25% as medium and 7.5% as low trustworthy [17]. A similar item was recorded in our study. A total of 91.7% of the patients examined here agreed or fully agreed with item 02 “I rate the data protection and data security of the questionnaire as high”, while only 8.4% did not agree or rather disagreed.

The influence of surrogate parameters of digital usage behaviour and basic epidemiological parameters, however, provides different results. For example, the studies by of Wong et al. and Suzuki et al. showed that elderly patients more often asked for support in filling out the questionnaire [19, 20]. According to Wong et al. the measured overall impression (“acceptance”) was also lower among the older study participants (see Table 6). In our study, an influence of age on the assessment of usability, comprehension of the questionnaire items and overall satisfaction with a tablet-based anamnesis questionnaire could not be demonstrated. Instead, the calculated mean value of the usability total score in the three defined age groups was 2.8 points and thus in the very high approval range. Wong et al. also measured the influence of computer skills on the overall impression (“acceptance”) of the digital questionnaire and were able to demonstrate that such influence was lower with 81% for low computer skills compared to 96% for high computer skills. The present study has also investigated this influence, but could not prove it with an overall high to very high level of the overall impression (“acceptance”). In the evaluation of usability on a scale of 0 to 10, an average of 8.0 points was rated in the group of patients with poor computer knowledge, 8.2 points in the group with moderate knowledge, 8.9 points in the group with good knowledge and 9.0 points in the group with moderate knowledge. Although a trend is evident in the scores, the difference between the groups was not significant in our study.

**Table 6:** Overview of the results of previous studies

Author	n	Average Age	Usability/Acceptance	Influence of age
Wong et al. [20]	121	58	92% „acceptable“ (dichotomous scale)	Acceptance lower among > 70 year olds (75% vs. 95%), more frequent requests for support (45% vs. 15%)
Schick-Makaroff et al. [18]	56	66	66%„very satisfied” 7% „satisfied” 2% „slightly satisfied” 18% „neutral”	Not proven
Suzuki et al. [19]	152	64	92% „easy to use“	Older patients needed support more frequently
Koch et al. [17]	80	47 (12–77)	80.25% „very satisfactory “ 19.75% „ satisfactory “ 1.25% „ unsatisfactory “	Not specified
Ferrari et al. [16]	74	37 *(rounded)*	97.3% „easy to use”	Not specified
Abernethy et al. [15]	66	55	98% “easy to use”	Not specified
Smith et al. [21]	150	44 *(rounded)*	Rated 1.3 on a six point scale (1 = very easy; 6 = very hard)	Not specified
Herrick et al. [22]	841	41 (18–95)	92% “very easy” to use	Older patients needed support more frequently
Benaroia et al. [23]	67	34	83% “very easy” to use	Not specified
Arora et al. [1]	173	–	93% easy to use	Not specified
Hess et al. [10]	10,999	47	84% “no difficulty”	Older patients were more likely to report difficulties with the usage
Weiner et al. [11]	82		93% very easy	Not specified

In summary, and in line with previous studies, it could be shown that patients across all ages and genders find digital questionnaires easy to use. No patient cancelled the questionnaire due to technical difficulties, while 93.0% of the patients stated that they had a positive overall impression regarding the digital anamnesis. All patients felt confident in using the digital questionnaire. The initial research hypothesis that older patients and patients with a low level of computer experience would experience problems filling out the digital questionnaire could not be confirmed. In addition, 91% of participants under 30, 95% of those aged 30–60 and 93% of those over 60 would recommend the digital questionnaire to other patients. Likewise, most patients perceived a digital questionnaire as suitable for presenting their medical concerns and reported feeling that a tablet questionnaire is safe in terms of data protection and security. Of all participants, 91.7% trusted the new technology and rated the type of data collection as safe, while 87.2% stated they would like to use a digital questionnaire again, and only 8.1% indicated a preference to fill out a paper sheet instead.

### Recommendations

The advantages and disadvantages of implementing a digital medical history survey in outpatient clinics must be carefully weighed up, however. The time saving promised by the use of digital questionnaires cannot be factored in directly, given that the treating doctor must (double) check the information themselves. In Germany, at least, obtaining the a patient’s history is defined by law as a procedure that must only be conducted by licensed physicians, and a questionnaire as such is only an aid in accomplishing this task [32]. Before the information has not been verified by the treating physician, no decisions may be made on the basis of the information collected, or it may even be entered in the patient file (such as: “The patient has no allergies”). There is at least a risk that this will occur, and checking an existing data record can also be an error-prone undertaking. In addition, it is important to note that, from a clinical perspective, a medical anamnesis is never just about the sheer collection of relevant information through direct questions. In addition to eliciting what is directly asked, the history should also provide meta-information about the psychosocial background, any personal or professional problems and the understanding of the illness. Non-verbal communication (i.e. for example facial expressions and gestures) can also provide valuable information about therapy planning. And finally, anamnesis simultaneously serves to establish a trusting doctor-patient relationship. In this context, digital anamnesis can only serve as a useful tool for the standardised collection of basic clinical information.

Large outpatient clinics might benefit the most from the implementation of digital questionnaires, given that these facilities likely experience the longest waiting times for patients due to busy intermediate processes (e.g. X-rays). However, the prerequisites for such an implementation should also be considered: The staff must be trained, and IT specialists and technical assistants are also required. In addition, licenses and necessary hardware must be purchased and theft protection must be established. It should also be considered that wireless communication may expose security risks in the internal network that might be covered by respective IT security specialists.

### Limitations of the study

The monocentric pilot study conducted has numerous limitations. The number of study participants is low with *n* = 86 patients, but in line with the other known studies, except for Herrick et al. and Hess et al. The largest number of patients was recorded by Hess et al. with *n* = 10,999, and the smallest by Schick-Makaroff et al. with *n* = 56 (see Table 6).

The age distribution of the study population was not normally distributed: Patients between 20 and 30 years of age were most frequent, while patients over the age of 60 were underrepresented. This limitation is problematic in that the influence of age on the measured scales of usability, content and overall satisfaction cannot be reliably answered for the actual population. The reason for this outcome could be a selection bias and/or an insufficient sample size. Although the patients were not selected based on epidemiological parameters, but rather on the basis of their presence, a subconscious preference when several inclusion-compatible patients were present at the same time is conceivable.

Furthermore, patients with mental or physical limitations (blindness, limited finger motor skills, etc.) that rendered them unable to understand or fill out the questionnaire were not included in the study. It can be assumed here that acceptance and use of digital consumer devices may be lower than in the rest of the study population. To address this issue, appropriately trained personnel should always be present in the waiting area, providing assistance if necessary. In addition, we did not use a control group to investigate possible difference in the overall impression and data quality between the digital questionnaire and the conventional paper-and-pen variant as the evaluation was based on a digital questionnaire.

In other known studies (see Table 6), supplementing influencing factors were also recorded. These included the level or frequency of assistance, the level of education (categorized by year) and the socio-economic status based on income. Our study did not aim to evaluate the medical information provided by patients in terms of truthfulness, quantity and quality.

## Conclusions

Given the advances in digital data collection and processing, during times of high demand in emergency rooms, strategies should be evaluated which utilize the waiting time of patients as efficiently as possible. Using a digital questionnaire in the waiting area of emergency departments and outpatient clinics might represent an innovative solution for this challenge.

This work demonstrated that patients across all ages and genders are confident in filling out a digital questionnaire as means of self-assessment of their medical history and their medical concerns of their emergency department visit.

The following conclusions can be drawn based on the above considerations: The digital anamnesis is a suitable instrument for collecting medical data. The assumption that older patients would experience difficulties in the operation could not be confirmed. The overall satisfaction of patients is high, regardless of the measured basic epidemiological factors and surrogate parameters of digital usage behaviour. A routine use of such software is conceivable. However, it still needs to be examined to what extent resources can be saved through the implementation of such digital questionnaires. In addition, the acceptance of this data collection by doctors should be evaluated. In further studies, we therefore plan to investigate under which conditions the digital anamnesis is perceived as valuable from a practitioner’s point of view.

## Supplementary Information

**Additional file 1.**

## Data Availability

The datasets generated and/or analysed during the current study are not publicly available due to patient privacy restrictions but are available from the corresponding author on reasonable request.
