# Supplementary material for: A pilot study of patient satisfaction with a self-completed tablet-based digital questionnaire for collecting the patient’s medical history in an emergency department
Source: BMC Health Serv Res. 2021 Jul 30;21:755. doi: 10.1186/s12913-021-06748-y (PMC8323085; doi:10.1186/s12913-021-06748-y)
Supplement: Supplementary file 1 — Additional file 1. [file 12913_2021_6748_MOESM1_ESM.pdf]

A pilot study of patient satisfaction with a self-completed tablet-based digital questionnaire for collecting the patient's medical history in an emergency department

Leander Melms[1,2*], Juergen R. Schaefer[1], Andreas Jerrentrup[1,3], and Tobias Mueller[1]

1 Centre for undiagnosed and rare diseases, University Hospital Gießen and Marburg, Marburg, 35033, Germany

2 Institute of Artificial Intelligence, Philipps-University Marburg, Marburg, 35033, Germany

3 Emergency Department, University Hospital Gießen and Marburg, Marburg, 35033, Germany

[*] Leander.Melms [at] uni-marburg [dot] de

Evaluation Survey I

01 How old are you?
- FREE TEXT [Numerical Input]

02 sex?
- Male
- Female
- Not specified

03 Mother tongue?
- FREE TEXT

04 Which devices do you use regularly in your private life?
- Mobile phone
- Internet-capable mobile phone / smartphone
- Tablet PC (Tablet)
- Stationary / fixed computer
- None of these

05 I consider my computer skills to be very good.
- Strongly agree
- Rather agree
- Rather not agree
- Strongly disagree

06 What visual impairment do you have?
- Myopia
- Long-sightedness
- None

07 Do you need a visual aid (e.g. glasses, contact lenses)?
- Yes
- No

08 Do you have any of the listed eye diseases?
- Colour blindness
- Macular Degeneration
- Glaucoma / Glaucoma
- Cataract / Glaucoma
- Diabetic retinopathy
- None

09 Do you have any of the listed motor limitations?
- Parkinson's Syndrome
- Rheumatic disease
- Amputations of fingers
- Numbness of the fingers
- Other
- None

Evaluation Survey II
- 01 The tablet was easy for me to use.
- 02 I rate the data protection and data security of the questionnaire as high.
- 03 The questionnaire has helped to clarify my medical concerns.
- 04 The (digital) questionnaire was usually quick to respond.
- 05 The questionnaire was clearly structured.
- 06 The answers to the questions worked perfectly from a technical point of view.
- 07 The time to complete the questionnaire was just right.
- 08 I had no difficulty in understanding the questions.
- 09 The questions seemed appropriate and conclusive to me.
- 10 The handling of the device was easy for me.
- 11 The font was legible.
- 12 The font size was not too small.
- 13 I felt confident to be able filling out the digital questionnaire.
- 14 I feel confident to be able filling out another digital questionnaire in the same format.
- 15 I had the impression that the questionnaire helps to sort my thoughts.
- 16 I was not afraid to damage the device.
- 17 I had no problems understanding the questions, even if medical terms were used.
- 18 I would rather not have filled out a paper and pen version of the questionnaire.
- 19 I was able to fill out everything with the questionnaire in peace and quiet.
- 20 I always knew exactly where to click to answer the questions.
- 21 I always knew what the questions were about
- 22 I would like to have a digital questionnaire again in the future
- 23 I would recommend the digital questionnaire.
- 24 My overall impression is quite positive
- 25 I was completely satisfied with the colour representation of the digital questionnaire
- 26 Unknown terms, if any, were explained
- 27 How satisfied were you with the usability of the digital questionnaire?

Emergency Survey Traumatology

What complaints do you bring to us?
- FREE TEXT

Are you in pain?
- Yes
- No

Where's the pain?
- PICTURE

How bad is the pain?
- POINTS (0 - 10)

When did the pain start?
- since a few days ago
- Suddenly, for several minutes/hours
- for several weeks

How long does the pain last?
- Constant pain with strong fluctuations
- Constant pain with slight fluctuations
- Pain attacks, painless in between
- Pain attacks, in between continuous pain

What character does the pain have?
- pulling
- piercing
- Pressing
- burning
- tearing
- oppressive
- cutting

Is the pain radiating?
- Yes
- No

Where does the pain radiate to?
- Into the chest
- in the legs
- in the shoulder
- in your arms
- at the back
- to the upper abdomen
- in the lower abdomen
- Belt-shaped

Are you taking medication regularly or currently?
- Painkillers
- anticoagulants like Marcumar, Aspirin, Plavix, Xarelto, Pradaxa, Eliquis, Lixiana, Heprarin
- Cardiovascular Drugs
- Antidiabetics especially metformin-containing
- Sleeping pills or tranquilizers
- Hormone preparations

Is there an allergy like hay fever or an allergic asthma or an intolerance of certain substances?
- Iodine
- Pollen
- X-ray contrast medium
- Patch
- Latex
- Drugs

Do infections occur frequently?
- No
- Yes

Do you have frequent bleeding symptoms (e.g. repeated spots on your arms/legs)?
- No
- Yes

Is there a postthrombotic syndrome?
- No
- Yes

Is there a cardiovascular disease?
- Heart attack
- Rhythm disturbances
- high blood pressure
- angina pectoris
- Heart failure

Does shortness of breath occur when climbing stairs?
- No
- Yes

Does a thyroid gland disease exist?
- Sub-function
- Goiter
- Overfunction

Is there a mental illness (depression, borderline syndrome)?
- No
- Yes

Is there an eye disease (cataract, glaucoma)?

- No
- Yes

Are there implants in the body?
- Joint Endoprosthesis
- Metal
- Silicone
- Stent
- Plastics

Has there ever been a scar proliferation like keloid?
- No
- Yes

Has an operation ever been performed, especially on bones, joints, ligaments, tendons or muscles?
- No
- Yes

If so, which ones?
- FREE TEXT

If so, were there any complications (bone/joint infection, healing disorders, circulatory problems, soft tissue calcification)?
- No
- Yes

Has the body area in question already been x-rayed or have examinations been carried out using other imaging methods (ultrasound, nuclear spin)?
- Yes
- No

Have you been vaccinated recently? If so, which ones?
- Tetanus
- Tytanus
- Flu vaccination

Could you be pregnant?
- No
- Yes

Are you breastfeeding?
- No
- Yes

Is there an allergy/sensitivity to metals?
- Nickel
- D-Penicillamine

Is there an acute inflammation or infection?

- Cold
- Fungal infection
- inflammatory skin disease

Is there an infectious disease?
- Yes
- No

Is there vascular disease?
- Coronary heart disease
- other vascular disease
- Arteriosclerosis

Has there ever been a vascular occlusion due to blood clots?
- Yes
- No

Are there or have there been thromboses or vascular diseases in the family (parents, siblings)?
- No
- Yes

If so, with whom and what illness?
- FREE TEXT

Is there low blood pressure or dizziness?
- No
- Low blood pressure
- Swindle

Is there a disease of the liver, gallbladder/bladder?
- No
- Gallstones
- Ignition
- fatty liver

Is there a disease or malformation of the kidneys or urinary organs?
- No
- Kidney stones
- Nephritis
- Renal dysfunction
- Bladder emptying fault

Is there a metabolic disease?
- No
- Diabetes
- Other metabolic disease
- Gout

Does a disease of the nervous system exist?

- No
- chronic pains
- Varicose disorders/epilepsy

Are there other diseases? If so, which ones?
- FREE TEXT

Regular smoking? If so, how much?
- FREE TEXT [Numerical Input]

Regular consumption of alcohol? If so, what and how much?
- FREE TEXT

Emergency Survey Internal Medicine

What complaints do you bring to us?
- FREE TEXT

Are you in pain?
- Yes
- No

Where's the pain?
- IMAGE QUESTIONNAIRE

How bad is the pain?
- POINT QUESTION (0 - 10)

Is the pain radiating?
- Yes
- No

Where does the pain radiate to?
- into the jaw
- in the lower abdomen
- Belt-shaped
- in your arms
- in the shoulder
- in the chest
- in the groin
- in the legs
- to the upper abdomen
- at the back

When did the pain start?
- since a few days ago
- for several years
- Suddenly, for several minutes/hours
- for several weeks
- for several months

How long does the pain last?
- Constant pain with strong fluctuations
- Constant pain with slight fluctuations
- Pain attacks, painless in between
- Pain attacks, in between continuous pain

What character does the pain have?
- stabbing
- pulling
- tearing
- burning
- Pressing
- oppressive
- cutting

Do you have any other accompanying symptoms?
- Anxiety
- Fever
- Nausea
- Breaking of coffee grounds
- Diarrhoea
- Constipation
- Shortness of breath
- Loss of appetite
- Flatulence
- Vomiting
- blood in urine
- Heartburn

Are you known to have a cardiac defect / valve defect?
- No
- Yes

Have you had heart surgery (e.g. bypass surgery)?
- Yes
- No

Are you familiar with bronchial asthma?
- Yes
- No

What's your height?
- SPECIAL NUMERIC FIELD WITH INPUT ASSISTANCE UI

Have you ever had a heart attack before?
- Yes
- No

Has a cardiac catheterization ever been performed before?
- Yes
- No

Was a stent implanted?
- Yes
- No

Are you aware of any cardiac arrhythmias (e.g. atrial fibrillation)?
- Yes
- No

Has there ever been a pulmonary embolism before?
- No
- Yes

Have you been implanted with a pacemaker/defibrillator?
- Yes
- No

Do you have any known constrictions of the vessels (legs, carotid artery, etc.)?
- No
- Yes

Are or were you aware of kidney stones/urinary stones?
- No
- Yes

Which cancer is/was known to you?
- FREE TEXT

Do you have a history of heart failure/heart failure?
- No
- Yes

Are you known to have chronic obstructive pulmonary disease (COPD)?
- No
- Yes

Are you familiar with pulmonary fibrosis?
- No
- Yes

Has there ever been a vascular occlusion due to blood clots (thrombosis, embolism)?
- No
- Yes

Are you or have you been diagnosed with renal dysfunction?
- No
- Yes

Are you a dialysis/CAPD patient?
- No
- Yes

Is a bladder voiding disorder known?
- No
- Yes

Have you ever had a stroke before?
- No
- Yes

Do you have or have you been known to have a spasm/epilepsy?
- No
- Yes

Are you known to have diabetes?
- Yes
- No

Are you aware of any hyperthyroidism?
- No
- Yes

Are you familiar with a goiter/stromata?
- No
- Yes

Do you have an increased tendency to bleed (e.g. frequent nose and gums bleeding, bruises, bleeding after operations)?
- No
- Yes

Are you or have you been diagnosed with cancer?
- No
- Yes

Are you familiar with any of the following infectious diseases?
- HIV/AIDS
- hepatitis B
- No - none

Is there a malignant blood disease (e.g. blood cancer, plasmocytoma)?
- No
- Yes

Do you have anemia?
- No
- Yes

Do you have a history of cirrhosis of the liver?
- No
- Yes

Are you known to have hepatitis?
- No
- Yes

Are or were gallstones known to you?
- No
- Yes

Do you suffer from dysphagia?
- No
- Yes

Are you or have you been diagnosed with a stomach ulcer?
- No
- Yes

Are or were you aware of diverticula in the bowel?
- No
- Yes

Have you ever been operated on before?
- Yes
- No

Operation - What and when?
- FREE TEXT

Do you have high blood pressure?
- Yes
- No

Did one of your parents suffer a heart attack/stroke at a young age (younger than 50 years) or even die?
- No
- Yes

Are you aware of elevated cholesterol levels?
- Yes
- No

You smoke?
- Yes
- No

If so, how much do you smoke per day?
- FREE TEXT [Numerical Input]

If so, for how many years?
- FREE TEXT [Numerical Input]

Do you drink alcohol?
- Occasionally
- Yes
- No
- not any more

What do you consume?
- beer or wine
- Spirituous beverages
- Alcoholic mixed drinks

How much do you consume per day?
- FREE TEXT

How much do you consume per week?
- FREE TEXT

Are you taking or have you taken drugs?
- No
- Yes

If so, which one?
- FREE TEXT

In which countries/regions have you been in the last six months?
- Caribbean
- Southern Europe
- East Asia
- Middle East

If so, when?
- FREE TEXT

Which yes, which country?
- FREE TEXT

Did the complaints occur during or shortly (up to 4 weeks) after a stay abroad?
- No
- Yes

If so, what complaints?
- FREE TEXT

Are you currently pregnant?
- No
- Yes

Have you had any miscarriages?
- No
- Yes

When was the last gynecological examination?
- DATE FIELD

How heavy are you?
- SPECIAL NUMERIC FIELD WITH INPUT ASSISTANCE UI

How's your appetite?
- Normal
- Bad
- Well

Do you have pain/burning when you urinate?
- No

- Yes

Is the urine foamy?
- No
- Yes

What is the color of the urine?
- Clear
- Yellow
- Red

What is the shape of the bowel movement?
- Sausage-like, lumpy
- Single soft, smooth-edged lumps, easy to excrete
- Sausage-like with smooth surface
- Single, solid beads, difficult to excrete
- Liquid, without solid components

What is the color of the bowel movement?
- Brown
- Bright

Do you have a cough?
- No
- Yes

Is the cough accompanied by sputum?
- No
- Yes

Are you sleeping soundly?
- No
- Yes

Are you having trouble breathing?
- No
- Yes

Do you sweat more at night?
- Yes
- No

Have you noticed any fever or increased temperature?
- No
- Yes

Have you lost weight unintentionally lately?
- No
- Yes

Do you suffer from swollen legs (edema)?
- Yes
- No

Do you have any known allergies or intolerances?
- No
- Yes

Is an allergy/intolerance to medication known?
- No
- Yes

Is an allergy/intolerance to latex known?
- No
- Yes

Is an allergy/incompatibility to disinfectants known?
- No
- Yes

Is an allergy/intolerance to X-ray contrast medium known?
- No
- Yes

Is an allergy/intolerance to plasters known?
- No
- Yes

Is an allergy/ intolerance to food?
- No
- Yes

Allergy/intolerance to food - if yes, which ones?
- FREE TEXT

Is your vaccination card ready?
- No
- Yes

Do you have a list of your medication with you?
- Yes
- No

Can you name any medications you take?
- No
- Yes

Are there any medications that you only take when needed?

- No
- Yes

Are there any medications that you or your doctor recently stopped or changed?
- No
- Yes

Have you taken any antibiotics in the last three months?
- Yes
- No

Do you take medication that you buy at the pharmacy without a prescription from the doctor? For example, aspirin, laxatives, heartburn remedies.
- No
- Yes

Do you use eye drops? Ear drops? Nose drops? Nose sprays?
- No
- Yes

Are you taking any anticoagulant medication? For example, Marcumar, Aspirin, Plavix, Xarelto, Pradaxa, Eliquis, Heparin
- No
- Yes

Are you taking antidiabetics (e.g. metformin)?
- Yes
- No

Do you take hormone supplements? Thyroid medication, birth control pills...
- Yes
- No

Hormone preparations - if so, which ones?
- FREE TEXT

Antidiabetics - if so, which ones?
- FREE TEXT

Are you taking sleeping pills and/or sedatives?
- No
- Yes

Drugs - if so, in what strength/ how often?
- FREE TEXT

Do you live alone?
- Yes
- No

Are you married?
- No
- Yes

Do you live in a stable partnership or in a domestic community?
- No
- Yes

Do you live in a retirement or nursing home?
- No
- Yes

Do you have a care level or degree of care?
- Yes
- No

Have you noticed recently that you have had less interest or pleasure in your activities?
- No
- Yes

Have you been feeling depressed, melancholy or hopeless lately?
- No
- Yes

What is your profession?
- FREE TEXT

Do you avoid crowds, public places, travelling alone or far from home?
- No
- Yes

Have you felt affected by nervousness, anxiety, tension, panic attacks or excessive concern in the last few weeks?
- No
- Yes

Which infectious disease are you familiar with?
- FREE TEXT

Are you aware of any thyroid dysfunction?
- No
- Yes

How many children have you born?
- FREE TEXT [NUMERICAL]

Miscarriages - if so, how many?
- FREE TEXT [NUMERICAL]

What form of contraception do you use?
- Other
- Spiral
- Preservative

How often do you have to urinate each day?
- FREE TEXT [NUMERICAL]

How often do you have to urinate at night?
- FREE TEXT [NUMERICAL]

How often do you have to have bowel movements during the day?
- FREE TEXT [NUMERICAL]

How many litres do you drink a day?
- FREE TEXT [NUMERICAL]

What colour is the sputum?
- Clear
- Yellow
- Green

Weight lost - if yes, how many kg?
- FREE TEXT [NUMERICAL]

Weight lost - if yes, in which period?
- FREE TEXT [NUMERICAL]

Incompatibilities - if so, which ones?
- Food, animal hair, hay fever
- Penicillin
- Hay fever
- patches, anaesthetic
- Lactose
- Cat

Allergies/intolerances to medicines - if so, which ones?
- FREE TEXT

Drugs - if so, which ones?
- FREE TEXT

Medication - if yes, when was the last time you took it?
- FREE TEXT

Medication as needed - if so, which?
- FREE TEXT

Medications discontinued - if so, which ones?

- FREE TEXT

Drugs without prescription - if so, which ones?
- FREE TEXT

Drops or sprays - if so, how many drops - how often - into which eye/ear?
- FREE TEXT

Anticoagulant drugs - if so, which ones?
- FREE TEXT

Sleeping pills and/or sedatives - if so, which ones?
- FREE TEXT
